# Hormetic Effects of Bioactive Compounds from Foods, Beverages, and Food Dressing: The Potential Role in Spinal Cord Injury

**DOI:** 10.1155/2021/6615752

**Published:** 2021-02-27

**Authors:** Anna Lucia Fedullo, Mario Ciccotti, Paolo Giannotta, Federica Alviti, Marco Bernardi, Anna Raguzzini, Elisabetta Toti, Tommaso Sciarra, Ilaria Peluso

**Affiliations:** ^1^Research Centre for Food and Nutrition, Council for Agricultural Research and Economics (CREA-AN), Rome, Italy; ^2^Military Pharmaceutical Chemical Plant, Florence, Italy; ^3^Department of Anatomy, Histology, Forensic Medicine and Orthopedics, Board of Physical Medicine and Rehabilitation, Sapienza University of Rome, Rome, Italy; ^4^Department of Physiology and Pharmacology “V. Erspamer”, Sapienza University of Rome, Rome 00185, Italy; ^5^Joint Veteran Center, Scientific Department, Army Medical Center, Rome, Italy

## Abstract

Spinal cord injury (SCI) is a damage or trauma to the spinal cord resulting in a total or partial loss of motor and sensory function. SCI is characterized by a disequilibrium between the production of reactive oxygen species and the levels of antioxidant defences, causing oxidative stress and neuroinflammation. This review is aimed at highlighting the hormetic effects of some compounds from foods, beverages, and food dressing that are able to reduce oxidative stress in patients with SCI. Although curcumin, ginseng, and green tea have been proposed for SCI management, low levels of antioxidant vitamins have been reported in individuals with SCI. Mediterranean diet includes food rich in vitamins and antioxidants. Moreover, food dressing, including spices, herbs, and extra virgin olive oil (EVOO), contains multiple components with hormetic effects. The latter involves the activation of the nuclear factor erythroid-derived 2, consequently increasing the antioxidant enzymes and decreasing inflammation. Furthermore, EVOO improves the bioavailability of carotenoids and could be a delivery system for bioactive compounds. In conclusion, Mediterranean dressing in addition to plant foods can have an important effect on redox balance in individuals with SCI.

## 1. Introduction

A spinal cord injury (SCI) is a condition that significantly impairs an individual's functional status, quality of life, and social independence (disability). The SCI can be divided into two main categories: the more common traumatic SCI typically caused by external physical impact [1] and nontraumatic SCI [2]. The different sites and the size of SCI can cause variable degrees of impairment from partial loss of motor or sensory function to complete paralysis below the injured spinal cord level, loss of bowel and/or bladder control, autonomic dysfunction (including in high SCI autonomic dysreflexia), and exaggerated reflex activities, as well as pain [3–8]. Based on these impairments, the interaction with the environment determines the different degrees of disability consequent to SCI. Regardless of the cause, the pathophysiology of SCI is characterized by two stages: an initial primary injury, defined as the immediate effects of an injury to the spinal cord, and a secondary progressive and self-propagating stage, characterized by multiple cascades of biochemical events in which oxidative stress is a critical component causing further tissue loss and dysfunction [3, 9–15]. The second stage is characterized by an increased formation of reactive oxygen species (ROS) and consequently by oxidative stress [16, 17]. Skeletal muscle atrophy, as well as general deconditioning, and sedentary lifestyle, commonly observed in people with SCI, can influence oxidative stress and antioxidant capacity [18, 19]. Antioxidant-based interventions have been suggested to alleviate oxidative stress and therefore to improve health in individuals with SCI [17, 20, 21].

In this context, bioactive compounds from Mediterranean diet [22, 23], as well as from beverages and food dressing [22, 24], have been proposed as hormetins, improving antioxidant defences by an hormetic mechanism mediated by the activation of the nuclear factor erythroid-derived 2 (Nrf2) antioxidant response element (ARE) pathway [22]. Many dietary components of the Mediterranean diet, such as culinary herbs and spices, as well as extra virgin olive oil (EVOO) are rich in bioactive phytochemicals [25]. Moreover, epigallocatechin-3-gallate (EGCG) from green tea, activating the Nrf2-ARE [26], is among the flavonoids suggested for treatment of SCI [23]. The aim of the present work is to review the hormetic effects of bioactive compounds from foods, beverages, and food dressing (olive oil, spices, and herbs) to reduce oxidative stress in patients with SCI.

## 2. Oxidative Stress in Spinal Cord Injury

**Figure 1:**
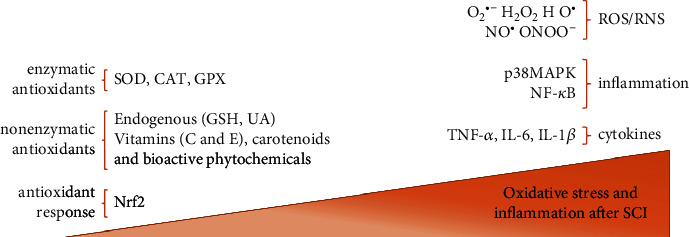
Representation of molecules involved in oxidative stress and inflammation after spinal cord injury (SCI). On the left are depicted as the antioxidants that are present in low concentrations, while on the right are molecules that are present at higher levels causing oxidative stress. SOD: superoxide dismutase; CAT: catalase; GPX: glutathione peroxidase; GSH: glutathione; UA: uric acid; Nrf2: nuclear factor erythroid-derived 2; O2^•−^: superoxide; H_2_O_2_: hydrogen peroxide; HO^•^: hydroxyl radical; NO^•^: nitric oxide; ONOO^−^: peroxynitrite; p38MAPK: p38 mitogen-activated protein kinase; NF-*κ*B: nuclear factor kappa-light-chain-enhancer of activated B cells; TNF: tumor necrosis factor; IL: interleukin.

Reactive nitrogen species (RNS) and ROS are produced continuously in the body, but an augmented production of ROS could exceed the capacity of the antioxidant defences (Figure 1), mediating in this way oxidative stress and subsequently oxidative damage [27, 28].

Superoxide (O_2_^•−^), produced by the mitochondrial electron transport chain, the xanthine oxidase (XO), and the NADPH oxidase (NOX), reacts with nitric oxide (NO^•^), produced by the nitric oxide synthase (NOS), to form peroxynitrite (ONOO^−^) [27, 29]. O_2_^•−^ can be converted to hydrogen peroxide (H_2_O_2_) by the superoxide dismutase (SOD). The isoforms of SOD include the copper(Cu)/zinc(Zn)-SOD localized in the cytosol and in the extracellular space and the manganese(Mn)-SOD localized in the mitochondria. In this context, Zn has an essential role as part of the antioxidant defence system. Little is known about the database on the Zn status and its time-dependent changes after SCI [30–33]. A predictive model for a long-term functional outcome was obtained analyzing Zn dynamics in 38 cervically injured SCI patients [32]. Heller and colleagues [33] investigated the dynamic alterations in serum Zn concentration during the first 72 h after injury in short intervals in order to identify the relationship between the early changes of the total Zn serum level and neurological impairment and patients' outcome. They found that the median Zn concentrations in the group with neurological impairment throw down within the first 9 h after injury stronger than those in patients with vertebral fractures without neurological impairment. They concluded that the outcome is related to early Zn concentration dynamics and may be considered a helpful diagnostic indicator for these patients. In fact, the changes in serum Zn levels allow an assessment of neurological impairment risk on the first day after trauma [33]. In this regard, it was shown that Zn treatment promoted motor function recovery during the 28 days following SCI and it seems to be able to reduce ROS and enhance the antioxidant activity [34].

Despite the antioxidant effect of SOD, in the presence of iron, H_2_O_2_ can generate via Fenton reaction the highly reactive hydroxyl radical (HO^•^), initiator of the lipid peroxidation.

Both catalase (CAT) and glutathione peroxidase (GPX) catalyze the conversion of H_2_O_2_ into water and oxygen [35]. Among endogenous antioxidants, the main enzymes are SOD, CAT, GPX, and glutathione reductase, while glutathione (GSH) and uric acid (UA) are the major nonenzymatic antioxidants [27] (Figure 1).

GSH acts as antioxidant by scavenging ROS through GPX and by the reversible oxidation to glutathione disulphide (GSSG). The latter is reduced to GSH by the glutathione reductase. On the other hand, although XO produces O_2_^•−^, it catalyzes the conversion of xanthine to UA which can scavenge O_2_^•−^ and HO^•^ and is the major antioxidant in body fluids and preserves neuronal viability in preclinical models of SCI [36]. GPX is a selenium- (Se-) dependent enzyme and it was shown that Se nanoparticles could reverse oxidative stress-induced SCI in rats [37]. Seelig et al. [38] recently compared Se, Cu, selenoprotein P, and ceruloplasmin levels in patients with traumatic SCI versus individuals with vertebral fractures without neurological impairment and found that Cu and Se levels at admission and Se and ceruloplasmin levels after 24 h were predictors for potential remission of SCI.

Among minerals, magnesium (Mg) is suspected to have a key role in the secondary injury phase. Low Mg serum levels within the first 7 days have been described to be correlated with high probability of neurological remission [39]. In particular, Mg appears to reduce the production of ROS and lipid peroxidation [40]. Markers of the lipid product of oxidation include 4-hydroxy-2-nonenal (HNE), alkenals, alkadienals, and malondialdehyde (MDA) being the thiobarbituric acid-reactive substances (TBARS) and F2-isoprostanes (F2-IsoP) derived by the nonenzymatic oxidation of polyunsaturated fatty acids [27].

Acrolein, an aldehyde produced endogenously through lipid peroxidation implicated in SCI, is more reactive than the other HNE and induces glutathione depletion [41]. On the other hand, Bastani et al. [42] analyzed a wide panel of antioxidant and oxidative stress biomarkers to define the antioxidant status in patients with SCI. They found that the urinary F2-IsoP and some enzymes (NOX and XO) in the vastus lateralis biopsies increased in the subjects with SCI compared with the controls, whereas SOD decreased. Besides, ROS production and apoptotic signals increased 1 and 3 months after SCI, while mitochondrial complexes and the SOD-2 protein content decreased 12 months after SCI [43].

On the other hand, advanced oxidation protein products (AOPP) in plasma, cerebrospinal fluid, and the spinal cord of rats increased after SCI and triggered generation of ROS (by activating NOX), with consequent induction of the p38 mitogen-activated protein kinase (p38MAPK) and the downstream regulated pathway nuclear translocation of nuclear factor kappa-light-chain-enhancer of activated B cells (NF-*κ*B) and proinflammatory cytokines [44]. AOPP include protein aggregates by disulphide bridges, as well as advanced peroxidation end products and advanced glycation end products. Other markers of protein oxidation include carbonyls [45] and the derivative of tyrosine from the reaction with the hypochlorous acid (HClO), generated by the H_2_O_2_-dependent reaction catalyzed by the myeloperoxidase (MPO) or with ONOO^−^ being 3-nitro-tyrosine as the main product of tyrosine oxidation [27]. Cysteine is particularly sensitive to oxidation and the reaction with NO^•^ produces S-nitrosylated cysteine, whereas in the presence of a proximal thiol group, ROS damage results in the formation of a disulfide bond [27]. Oxidation of cysteine residues could be an essential feature for signaling pathways, including Nrf2/ARE.

## 3. Dietary Antioxidants in Spinal Cord Injury

The dietary advice for individuals with SCI included Mediterranean diet [46] and an anti-inflammatory diet [47]. The latter was able to increase (after 3 months) the intake of vitamins C (ascorbic acid) and E (alpha-tocopherol) in individuals with SCI, where proinflammatory markers were negatively correlated with carotenoids [47]. Patients with this condition (from at least 2 years) showed lower serum levels of these vitamins [48] and of vitamin E and beta-carotene [49], compared with healthy controls. Vitamins (C and E) and several bioactive compounds (such as carotenoids, phenolic compounds, and glucosinolates) are exogenous antioxidants that account for the antioxidant capacity of dietary sources (Table 1).

In 1991, an innovative study, subsequently confirmed in [54, 55], proved for the first time that rats treated with vitamin E were protected against induced muscle atrophy [56]. Nevertheless, this protection seemed to be due to the downregulation of genes involved in the proteolysis of muscles, rather than by the antioxidant properties of vitamin E [57]. It has been reported that an improved bladder recovery and locomotor function in rats is associated with vitamin E-enriched diet. In fact, in order to improve sensory and autonomic dysfunctions associated with SCI, the potential use of vitamin E was suggested [58]. Moreover, vitamin E treatment markedly enhanced the hind limb locomotor function, reduced the histopathological alterations and the morphological damage in the spinal cord, and the lowered MDA level and GPX activity in SCI [59]. On the contrary, combined treatment of vitamins C and E significantly contrasted the effects of spinal cord contusion on oxidative stress, increasing SOD and GPX [60]. Recently, synergistic effects of vitamin C and taurine against SCI in rats have been investigated and the combined treatment decreased mRNA expression of interleukin- (IL-) 6, cyclooxygenase- (COX-) 2, tumor necrosis factor- (TNF-) *α*, and inducible NOS (iNOS) compared to the single treatments and recovered altered antioxidant markers [61]. Moreover, vitamin C treatment alone suppressed NF-*κ*B, COX-2, and iNOS expressions in renal tissue, reduced the inflammatory responses (TNF-*α* and IL-1*β*) and oxidative stress (TBARS, protein carbonyl, and MPO), and enhanced the antioxidant status (GSH, SOD, CAT, and GPX) after SCI-induced kidney damage [62]. On the other hand, the lipid-soluble plant pigments carotenoids, having antioxidant activity, have been suggested as neuroprotective nutraceuticals [63, 64]. The carotenoid lycopene found richly in red fruits and vegetables, due to its lipophilic structure, can pass through the blood-brain barrier and reach the brain [63]. It was demonstrated that lycopene treatment in SCI rats significantly improved oxidative stress, by reversing SOD, GPX, and MDA alterations [65]. Lycopene reduced lipid peroxidation in murine models [65, 66] and NF-*κ*B activation in a mouse model of SCI [66]. Similar inhibition of NF-*κ*B has been reported for beta-carotene in a rat model of SCI [67]. In particular, astaxanthin, crocetin, and lycopene decreased pain [68–72]. Moreover, astaxanthin [71], crocetin [73] and crocin improved locomotor function [74].

Among flavonoids, a study conducted in mice by Borghi et al. [75] showed that quercetin could be useful to treat muscle pain conditions linked to unaccustomed exercise due to its capacity to inhibit spinal cord cytokine production, oxidative stress, and glial cell activation. Furthermore, an experimental study conducted in rats by Ocal et al. [76] suggested that quercetin can be thought as an option of treatment in SCI. Quercetin [77] and the citrus flavonoid hesperidin [78] exerted an anti-inflammatory effect. Several studies showed that the administration of the stilbene resveratrol after SCI could provide a beneficial impact on the neurological recovery and the antioxidant activity in rats [79–83], and a recent meta-analysis of studies in rat models of SCI revealed that it increased SOD and decreased MDA levels, compared to the control group [84].

The food dressing-derived bioactive compound rosmarinic acid, identified in rosemary (987 mg/100 g) from which its name derives [25], has been suggested for SCI in a recent review [85], whereas antioxidant and/or anti-inflammatory activities in murine models of SCI have been reported for curcumin [86–88] and oleanolic acid [89].

## 4. The Nuclear Erythroid 2-Related Factor 2 as the Target for Spinal Cord Injury Treatment

Nrf2 is a transcription factor that regulates the antioxidant response system and inhibits oxidative stress-mediated NF-*κ*B activation by decreasing the intracellular ROS levels [90, 91]. Normally, Nrf-2 is localized into the cytoplasm bound to the Kelch-like ECH-associating protein 1 (Keap1) that contains cysteine residues sensitive to oxidants or electrophiles [27]. Upon oxidation, Keap1 forms a disulfide bond and the conformational change results in the release of Nrf-2, allowing its translocation into the nucleus. Nrf-2 promotes the transcription of target genes containing the ARE in their promoter regions, including antioxidant enzymes and heme oxygenase 1 (HO-1). HO-1 is among the Nrf2-induced genes that inhibit NF-*κ*B activation [90, 91]. NF-*κ*B is normally sequestered inactive in the cytoplasm of resting cells by the inhibitor *κ*B (I*κ*B). The phosphorylation of two serines of I*κ*B, by the I*κ*B kinase (IKK), and its subsequent degradation by proteasome allow the activation of NF-*κ*B and its translocation to the nucleus [90, 91]. After nuclear translocation, NF-*κ*B induces the expression of proinflammatory cytokines, as well as of ROS-producing enzymes, including COX-2 and iNOS [91]. Increasing levels of TNF-*α*, IL-6, COX-2, and iNOS activate the Nrf2/HO-1 axis that subsequently decreases their own expressions [91]. In addition, upregulation of Nrf2 reduces the I*κ*B-*α* proteasomal degradation and inhibits nuclear translocation of NF-*κ*B [91]. NF-*κ*B decreases the free CREB-binding protein (CBP also known as CREBBP), which is a transcriptional coactivator of Nrf2 by competing with CBP [91].

**Figure 2:**
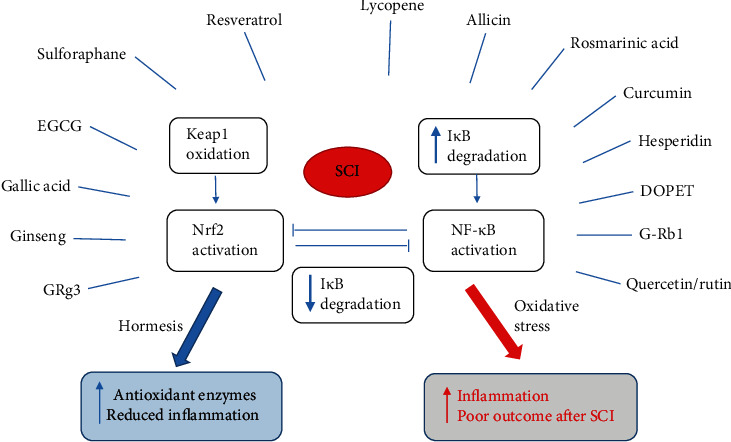
Bioactive compounds that act on the Nrf2/NF-*κ*B pathway. DOPET: 3,4-dihydroxyphenylethanol; EGCG: epigallocatechin-3-gallate; GR: ginsenoside R; I*κ*B: inhibitor *κ*B; Keap1: Kelch-like ECH-associating protein 1; NF-*κ*B: nuclear factor kappa-light-chain-enhancer of activated B cells; Nrf2: nuclear factor erythroid-derived 2; SCI: spinal cord injury.

On the other hand, antioxidants with electrophilic moieties induce the Nrf2-mediated gene expression of antioxidant enzymes acting as prooxidants rather than antioxidants [26, 27, 92]. Besides, electrophilic modifications of cysteine 179 of IKK inhibit NF-*κ*B activation and have been suggested as one of the mechanisms involved in the anti-inflammatory effects of nutraceuticals [27, 92]. Therefore, Nrf2 has a fundamental role in the hormetic effect of natural bioactive compounds (Figure 2) and its signal pathway crosstalk with the NF-*κ*B pathway in animal models of SCI [93].

Hormetins typical of Mediterranean diet include molecules that interact with these transcription factors, such as vitamin E and many phytochemicals (terpenoids, phenolic antioxidants, allium-derived sulfur compounds, carotenoids, and resveratrol) from grapes, fruits, tomatoes, leafy green vegetables, legumes, onion, garlic, olives [22], and EVOO [94]. On the other hand, nonnutrient phytochemicals from spices often used for culinary purposes, namely, curcumin and ginger, as well as herb extracts (green tea extract, ginseng-based steroids, and ginsenosides) showed the capability to improve both oxidative stress and the inflammatory status in humans [92]. Some of these have been studied as bioactive molecules potentially useful against neurodegenerative diseases such as SCI [95].

Curcumin increased SOD levels [96] and decreased MDA [96] and proinflammatory cytokines, like TNF-*α* and IL-1 [97], and exerted its neuroprotective effect through the crosstalk between NF-*κ*B and Nrf2 signaling pathways [97]. Similar effects on the NF-*κ*B/Nrf2 pathway have been reported for sulforaphane, an isothiocyanate derived from broccoli, that is a potent naturally occurring inducer of the Keap1/Nrf2/ARE pathway and could mitigate inflammation through the inhibition of the NF-*κ*B pathway [98]. Also, EGCG from green tea induces the Keap1/Nrf2/ARE pathway [26]. To investigate neuroprotective potential of green tea polyphenols, Zhao et al. [99] induced oxidative damage in spinal cord neurons using H_2_O_2_ and applied different concentrations of green tea polyphenols to the cell medium for 24 hours. Measurements of SOD activity and MDA content revealed that green tea polyphenols reduced oxidative stress [99].

Ginseng treatment significantly downregulated inflammatory markers and oxidative stress by enhancing the antioxidant status in SCI rats [100]. In particular, ginsenoside R (GR) b1 attenuates SCI-associated oxidative stress in rats by regulating the endothelial NOS/Nrf2/HO-1 signaling pathway and increased SOD, CAT, and GSH [101], whereas GR g3 show anti-inflammatory, antioxidant, and neuroprotective effects, suppressing mRNA expression of proinflammatory cytokines (TNF-*α*, IL-1*β*, and IL-6) and the overproduction of COX-2 and iNOS after SCI [102]. Reductions of COX-2 and NF-*κ*B expression have been observed also with gallic acid [103], a phenolic acid contained in various plant-food sources [53]. Hesperidin, a representative flavonoid in citrus fruits, reduced proinflammatory cytokines including TNF-*α* and IL-1*β*, whereas it increased SOD, CAT, Nfr2, and HO-1 [104].

It was shown that resveratrol treatment suppressed the activation of the iNOS/p38MAPK pathway and reduced oxidative stress by enhancing enzymatic and nonenzymatic antioxidant levels such as those of GSH, SOD, and CAT in spinal cord ischemia-reperfusion injury-induced rats [105]. Furthermore, resveratrol showed a neuroprotective effect by increasing the activation of Nrf2 [106]. Preclinical studies showed that the administration of resveratrol in the acute phase or prior to experimental injury to the central nervous system could have a neuroprotective [107]. Similar results were demonstrated for quercetin. In fact, in SCI rats, quercetin has protective effects on the spinal cord by the potential mechanism of inhibiting the activation of the iNOS/p38MAPK signaling pathway and thus regulating secondary oxidative stress [108]. Quercetin treatment reversed MDA, NO, MPO, and cytokine levels and banned the exhaustion of tissue GSH levels and SOD [109]. Also, the quercetin-3-O-rutinoside (rutin) exerts neuroprotective effects through anti-inflammatory inhibition of the p38MAPK pathway [110]. A good source of quercetin is onion [53], and among bioactive compounds from Mediterranean food dressing, there are also rosmarinic acid, allicin, and 3,4-dihydroxyphenylethanol (DOPET).

Rosmarinic acid is a water-soluble polyphenolic phytochemical that could enhance the antioxidant status and consequently decrease the oxidative stress in Wistar rats post-SCI by targeting Nrf2/HO-1 and NF-*κ*B pathways, downregulating proinflammatory cytokines (TNF-*α*, IL-6, and IL-1*β*), and acting as neuroprotective agent [111, 112].

Garlic and onion are rich in organosulfur compounds, including allium and allicin, that induce the Nrf2 pathway [22]. Allicin, the main biologically active compound derived from garlic, seems to have neuroprotective effects in animal models, being able to increase the activities of antioxidant enzymes, including CAT, SOD, GPX, and glutathione S-transferase [113]. In addition, it was shown that allicin enhanced the motor functional recovery and increased Nrf2 nuclear expression [114], while it decreased the expression of inducible NOS but had no effects on the expression of neuronal NOS following glutamate exposure [115].

DOPET is a potent antioxidant polyphenolic compound from EVOO targeting multiple signaling pathways to reduce SCI effects, including reduction of MPO and downregulation of proinflammatory cytokines [116]. Moreover, it has been previously reviewed that other bioactive compounds of EVOO, such as hydroxytyrosol [22, 94] and oleuropein [22], can activate the Nrf2 pathway, but specific studies are needed on SCI. Transcription of antioxidant genes mediated by Nrf2 could be also enhanced by ferulic acid (present in fruit, tomatoes, and rice), luteolin (present in carrots, peppers, and celery), phenethyl isothiocyanate (present in crucifer vegetables), and carnosic acid (abundant in rosemary) [22]. Therefore, many bioactive compounds should be tested in SCI in future studies.

## 5. Conclusion

SCI results, since the early stages, in an imbalance between the ROS production and antioxidant defences (Figure 1). Low levels of some micronutrients, including antioxidant vitamins and minerals involved in antioxidant enzymes' activity, have been reported in individuals with SCI. ESPEN guidelines suggested supplementation with antioxidant micronutrients for patients in the intensive care unit [117] and with neurological diseases [118] and reported in an observational study that this is practiced also in individuals with cervical SCI [119].

Dietary advice and supplements have been proposed in order to reduce oxidative stress, and in some cases, synergistic effects have been reported. Although curcumin, ginseng, and green tea have been proposed for SCI management, low levels of antioxidant vitamins have been reported in individuals with SCI. Mediterranean diet that includes food, spices, and herbs contains multiple components with antioxidant properties (Table 1), such as vitamins, phenolic compounds, and glucosinolates. The latter are known to activate Nrf2 by an electrophilic interaction with sulfhydryl-groups on Keap1, therefore in a hormetic manner. Oxidation of cysteine residue of Keap1 is involved in the EGCG induction of Nrf2. On the other hand, nonnutrient bioactive compounds from food, spices, and herbs typical of the Mediterranean diet could reduce oxidative stress by activating the Nrf2 pathway, acting as hormetins. Although many of these compounds have low bioavailability, hormetic effects typically occur at low concentration. Moreover, nanoparticle-based formulations have been suggested to improve bioavailability of flavonoids [120] and carotenoids [121] and resveratrol efficacy in SCI [122]. In particular, rats with SCI treated with resveratrol- and puerarin-loaded nanoparticles showed a decrease of GSH, SOD, and CAT antioxidant levels [122]. On the other hand, squalene from EVOO has been suggested as natural delivery system for bioactive compounds [123]. It was observed that carotenoids' absorption was higher in people that consumed salads with full-fat dressing [124]. Furthermore, EVOO is a source of vitamin E and contains many bioactive compounds [22]. From that, Mediterranean dressing in addition to plant foods can have an important effect on the redox balance in individuals with SCI. From a clinical point of view, this evidence could support the patients during both the early rehabilitation phases and the chronic management. In conclusion, the previously suggested hormetic effects of Mediterranean diet [22] that can be considered a natural multicomponent supplement [125] could be useful for the long-term management of SCI.

## Figures and Tables

**Table 1:** Some common sources of antioxidants of the Mediterranean diet.

	Glucosinolates (mg/100 g)	Vitamin C (mg/100 g)	Vitamin E (mg/100 g)	Retinol equivalents (*μ*g/100 g)	Beta-carotene (*μ*g/100 g)	Total phenolics^∗^ (mg/100 g)
Broccoli	61.7	77	1.3	123	738	89
Brussels sprouts	236.6	81	1.0	220	1320	221
Cabbage	58.9	47	0.18	19	738	81.73
Cauliflower	43.2	59	0.15	50	114	88.63
EVOO		—	22.4	36	—	55.14
Garlic		9	—	1	6.9	87.04
Kale	100.7	110	2.24	225	1350	176.67
Onion		5	0.22	3	0	69.49
Parsley		162	1.29	943	5658	836.9
Radish	92.5	18	0	0	0	44.3
Rosemary		29	1.5	92	550	1212.3
Sage leaves		0	9.15	215	3540	1049.3
Turnip	93.0	23	2.44	0	1794	93.5

^∗^Folin assay. Data from [50–53].

## Abbreviations

AOPP: Advanced oxidation protein products
ARE: Antioxidant response elements
CAT: Catalase
COX: Cyclooxygenase
Cu: Copper
DOPET: 3,4-Dihydroxyphenylethanol
EGCG: Epigallocatechin-3-gallate
EVOO: Extra virgin olive oil
F2-isoP: F2 isoprostanes
GPX: Glutathione peroxidase
GR: Ginsenoside R
GSH: Glutathione
GSSG: Glutathione disulphide
H_2_O_2_: Hydrogen peroxide
HClO: Hypochlorous acid
HNE: 4-Hydroxy-2-nonenal
HO: Heme oxygenase
HO^•^: Hydroxyl radical
IL: Interleukin
I*κ*B: Inhibitor *κ*B
IKK: I*κ*B kinase
iNOS: Inducible NOS
Keap1: Kelch-like ECH-associating protein 1
MAPK: Mitogen-activated protein kinase
MDA: Malondialdehyde
Mg: Magnesium
Mn: Manganese
MPO: Myeloperoxidase
NF-*κ*B: Nuclear factor kappa-light-chain-enhancer of activated B cells
NO^•^: Nitric oxide
NOS: Nitric oxide synthase
NOX: NADPH oxidase
Nrf2: Nuclear factor erythroid-derived 2
O2^•−^: Superoxide
ONOO^−^: Peroxynitrite
RNS: Reactive nitrogen species
ROS: Reactive oxygen species
SCI: Spinal cord injury
Se: Selenium
SOD: Superoxide dismutase
TBARS: Thiobarbituric acid-reactive substances
TNF: Tumor necrosis factor
UA: Uric acid
XO: Xanthine oxidase
Zn: Zinc.
